# The role of community energy in mediating sustainable energy transitions in East and Southern Eastern Africa

**DOI:** 10.1038/s44406-026-00024-w

**Published:** 2026-05-20

**Authors:** Vanesa Castán Broto, Mulualem Gebreslassie, Getachew Bekele, Amare Assefa, Adugnaw Lake, Dawit Habtu, Solomon T. Bahta, Fana Filli Nurhussien, Chrispin Gogoda, Christopher Hara, Lorraine Howe, Carlos Shenga, Harshit Vallecha, Akatew Haile Mebrahtu

**Affiliations:** 1 University of Sheffield, Sheffield, UK; 2 Sheffield Hallam University, Sheffield, UK; 3 Addis Ababa University, Addis Ababa, Ethiopia; 4 Mekelle University, Mekelle, Ethiopia; 5 Mzuzu University, Mzuzu, Malawi; 6 Centre for Research on Governance and Development (CPGD), Maputo, Mozambique

**Keywords:** Environmental sciences, Environmental social sciences, Energy and society, Environmental studies

## Abstract

Community energy projects, actively governed and managed by community members, play an essential role in advancing just energy transitions, for example, by providing energy in remote areas, facilitating the adoption of renewable technologies, and building resilient electricity networks. However, their adoption remains limited. How does community energy work in practice, and how can it become more widespread? This article presents a study of community energy in three countries: Ethiopia, Malawi, and Mozambique. A multi-methods analysis (project inventories, qualitative interviewing, surveys) suggests that the political economy of development and material challenges, such as financing and supply chains, constrain the expansion of community energy. A survey of community energy beneficiaries demonstrates the tangible benefits these projects bring to disadvantaged communities. Concessional grants that recognise the social value of community energy can facilitate its development and support new energy models for a just energy transition.

## Introduction

Community energy refers to energy systems in which people play a key role in governing and defining energy uses^1–4^. These systems effectively put people at the centre of sustainable energy transitions. At the same time, community energy can play an essential role in achieving Sustainable Development Goal 7, which aims to “Ensure access to affordable, reliable, sustainable and modern energy for all.” In remote areas, for example, community energy systems may facilitate the implementation of off-grid technologies independent from the grid. In urban areas, where people often live ‘under the network’ without access to electricity for socio-economic reasons (affordability, lack of access), community energy can mediate collective services^5^.

Advancing sustainable energy transitions requires *putting people at the centre* of infrastructure transformations, as it:

- Improves the effectiveness of projects that advance the energy transition. Business models for managing off-grid systems facilitate electricity generation near where it is used^6^.
- Facilitates the spread of technologies. Active project engagement helps people understand renewable technologies and maintain them more effectively^7,8^. People working in community energy often become advocates of renewable technologies and help develop the skills force^9,10^.
- Facilitates a just transition to sustainable energy. Renewable energy projects are not intrinsically good: they may negatively impact communities if they fail to account for their needs. When people lead community energy projects, they identify appropriate needs and negative impacts^11^.

Despite encouraging initiatives across the African continent, several barriers hinder the advancement of community energy initiatives. Limited resources and technologies can discourage advocates in this field by preventing them from securing financial resources^12^. Additionally, communities struggle to comprehend projects or lack the cohesion needed to manage them effectively^13,14^. Successful projects also face challenges, including a lack of emergency funds for post-disaster repairs or insufficient capacity for ongoing maintenance^15^. Furthermore, community energy projects rely on broader networks that influence communities, such as political and economic institutions.

Case study analyses have demonstrated the local potential with communities providing resources such as effective governance systems and embedded notions of environmental stewardship^16^. Given the enormous gap in human resources for the energy transition, mobilising local skills and capacities is an essential strategy to facilitate the adoption of renewables^17^. Comprehensive capacity-building programmes are called for in the development of renewable energy. However, communities may find themselves alone, unable to access further resources. To address this knowledge gap, our research team aimed to develop a comparative analysis of challenges across different projects and national political contexts.

To our knowledge, this paper presents the first comprehensive investigation of how the political context, finance, technology supply chain, and community dynamics impact the development of community energy initiatives from a comparative perspective, focusing on the current state of the sector in Ethiopia, Malawi, and Mozambique. The paper foregrounds the experiences of project managers and users. Empirical evidence shows that community energy plays a central role in advancing an inclusive energy transition in these countries, but it needs institutional support to overcome current challenges.

The focus of this paper is on understanding how community energy works in practice and how it can become more widespread. On this basis, the paper asks three specific questions that structure the presentation of the results:

- How does each country’s political and governance context shape community energy projects and facilitate their integration in the energy transition?
- What are the material and institutional conditions that shape the success of community energy projects?
- How does community energy contribute to facilitating just energy transitions, particularly through providing benefits to disadvantaged groups who lack energy access?

Following these questions, the following section presents the empirical results of a 4-year international project that studied the development of community energy in Ethiopia, Malawi and Mozambique. The project compiled a wealth of empirical evidence across the three countries, engaging directly with the development and maintenance of existing projects (see Section “Methods” for a detailed description of data collection methods). Based on a holistic interpretation of empirical data, “Results” presents the results in three sections that respond to the questions above. “Discussion” presents a discussion of results, conclusions and policy recommendations.

## Results

### How the political and governance context shapes community energy

In Ethiopia, the National Electrification Program (NEP), now in its second iteration (NEP2), is the primary tool for advancing electrification plans, combining on-grid expansion with off-grid, locally tailored projects^18^. The federal institutions responsible for electrification are the Ministry of Water and Energy (MoWE), the Ethiopian Energy Authority (EEA), and the Ethiopian Electric Power (EEP). While there is an emphasis on public-led infrastructure development in Ethiopia, since 2020, NEP2 has included measures to facilitate the entry of Independent Power Producers (IPPs). The policy emphasis is on stabilisation, green growth and the development of a sustainable energy mix. In 2024, MoWE developed the National Sustainable Energy Development Strategy (N‑SEDS) 2024–2030 in consultation with relevant private-sector and civil-society stakeholders to facilitate near-universal energy access through a just and inclusive transition. N-SEDS emphasises off-grid energy not only as providing energy access in remote areas (as per NEP2) but also as providing alternatives to hydropower (now threatened also by climate change-exacerbated droughts) and facilitating the entry of private capital. However, the implementation of these policies is marked by a culture of state-led modernisation and tight political control, in line with the authoritarian developmentalism that dominates the country’s development: the same political culture that exacerbates the dynamics of exclusion across its different regions, and the inter-ethnic tensions leading to conflicts such as the Tigray Civil War (2020–2022)^19^.

The energy transition in Malawi is marked by extremely low rates of electricity access and high dependence on biomass, which the government has been trying to curtail through regulations to prevent deforestation. The main framework for the country’s energy sector is the National Energy Policy (2018), which emphasises affordability and reliability. Key institutions in the energy sector are the Malawi Energy Regulatory Authority (MERA), the Department of Energy Affairs (DoEA), and the Electricity Supply Corporation of Malawi (ESCOM). Off-grid development has been supported since 2017 by the Malawi Renewable Energy Strategy, and governmental institutions have been favourable not only to private-sector participation but also to the establishment of cooperatives and other community-led projects supported by varied sources of ODA finance. However, slow implementation shows gaps between vision and practice, particularly in the context of high FOREX dependency. The latest attempt to consolidate Malawi’s approach to the energy transition is the 2025 National Energy Compact for Malawi, which is part of Mission 300, a continental initiative from the World Bank and African Development Bank (AfDB) to bring electricity to 300 million Africans by 2030. A key innovation of the Compact is the coupling of expanding access to renewables with facilitating access to clean cooking (which is an aspect of energy access that tends to be overlooked in favour of lighting, communications, and productive uses of energy).

Mozambique has a well-developed energy transition strategy with a set of combined regulations that aim to achieve universal energy access by 2030, a diversified energy mix to reduce dependence on fossil fuels and hydropower, the entry of independent power operators to facilitate private-sector investment, and social inclusion. The primary tool to achieve this is the *National Electrification Strategy (ENE) – Energy for All* (2018-2030). ENE consolidated the complex institutional structure of the energy sector, including organisations such as the Ministry of Mineral Resources and Energy (MIREME) in charge of planning; the regulatory agency ARENE; the public utility Electricidade de Moçambique (EDM) in charge of grid-based expansion; the Energy Fund (FUNAE), responsible for rural and off-grid electrification; and the Ministry of Economy and Finance that allocates funding. In 2023, MIREME presented the Just Energy Transition Strategy at COP28, following previous policies of renewable development and grounded on a Regulation for Energy Access in Off-Grid Areas (Decree 93/2021) and a new electricity law in 2022 (Law 12/2022). However, despite this advanced framework for energy transitions, the energy sector remains tied to extractive industries, which attract international investments, allegations of corruption, and citizens’ continued dependence on charcoal and firewood^20^. In 2021, stakeholders welcomed the new off-grid regulation to accelerate the entry of IPPs to energy markets, and implementation frameworks have been amended to facilitate the approval of smaller energy projects. However, government control over supply chains and investments hinders the development of new projects. ODA investments have accounted for the bulk of off-grid development, with programmes such as Brilho, funded by the UK Foreign, Commonwealth & Development Office (FCDO) (formerly DFID) and implemented by the SNV-Netherlands Development Organisation.

**Fig. 1:**
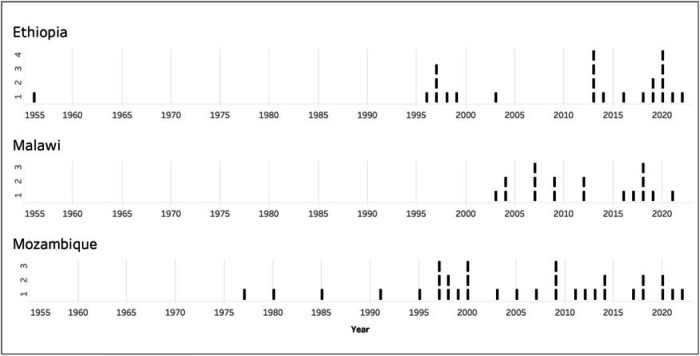
Count of national energy policies and regulations over time (1956–2022). The figure illustrates the cumulative adoption of energy-sector policies and regulations at the national level in Ethiopia, Malawi, and Mozambique between 1956 and 2022. It highlights the progressive expansion of formal policy frameworks governing the energy sector in each country. Source^21^.

In each country, the potential of community energy to catalyse an energy transition depends on existing electrification policies and the alignment of regulatory frameworks with implementation practices. Our work over three years (2019–2022) examined data on the development of energy legislation in the three countries, which we summarise in Fig. 1. Mozambique has the most extensive energy legislation and regulations (32 items), followed by Ethiopia with a moderately concentrated body (23 items), and Malawi with the least extensive body (17 items). Malawi’s energy legislation spans from 2003 to 2021, showing a more concentrated energy policy. In contrast, Ethiopia has two concentrated periods of energy policy (1996–1999 and 2013–2022) while Mozambique’s energy policy is more dispersed (Fig. 1).

Statistical analysis found that an extensive body of energy legislation and regulation correlates with greater rates of electricity access than a less extensive one^21^. While the number of laws may not be a sufficient measure, the quality and scope of the regulatory framework are critical in enabling smaller-scale, community-led energy projects^21^. However, project delivery remains a challenge, even when a consistent body of regulation exists. Bureaucratic and complex permitting processes hinder community groups from obtaining the necessary licenses for energy projects and may also impede access to land. Following the quantitative analysis of the regulatory landscape, the research team examined operators’ and policymakers’ perceptions of the realities of community energy.

The first challenge is the lack of accommodation of community energy actors within the existing institutional landscape in the energy sector. In each country, the unbundling of responsibilities across different institutions has led to institutional fragmentation. Institutions accustomed to operating with centralised networks struggle to accommodate the needs of small operators such as those in charge of community energy, often discouraging incipient projects. Licensing processes may be delayed due to a lack of coordination across agencies. For example, in Ethiopia, coordination challenges involve MoWE, the EEA, and the EEP, all of which hold central responsibilities within NEP2. In Malawi, multiple institutions oversee energy policies, including MERA, DoEA, and ESCOM. Still, there is little collaboration between them, which creates additional regulatory uncertainty. In Mozambique, mini-grid developers must go through multiple approval stages from MIREME, ARENE, and local governments. However, the bulk of experience in developing off-grid projects remains with FUNAE, whose knowledge legacy is not consistently recognised in the rapidly evolving landscape of off-grid development.

The lack of policy alignment and coordination among institutions and stakeholders presents a key barrier to scaling up community energy development. There is a gap between the approval of ambitious national plans and their implementation in remote regions. The operational legacies of centralised electricity systems loom large in the implementation of new renewable technologies, even with policies to facilitate the adoption of off-grid models. Regions often face a gap in institutional capacity to enforce such plans, particularly in terms of transforming existing practices to enable community energy. For example, in Ethiopia, a senior expert at the Energy Agency in Tigray explained that “*the federal government provides the mandate for regions to develop their own decentralised off-grid energy systems through the off-grid renewable energy development plan*” (EM1-Ethiopia). Similarly, other experts in both the Tigray and Sidama regions emphasise that new policies have enabled regional authorities to respond flexibly. Yet, regulatory codes and guidance may not align with stated policy goals. In Malawi, for example, a project manager explained that “*the government encourages renewable mini-grids, but there are no clear policy guidelines for licensing community-run hydro systems*” (EM9-Malawi). And while from an international policy-making perspective, such as from a representative of a multilateral bank (EM17-Mozambique), regulations reduce grey areas and give confidence to investors, in practice, there is an inherent uncertainty and additional time needed for developing “*accessory regulations*” (EM16-Mozambique), which further delays implementation.

The consequence felt at the local level is complex licensing systems, as various agencies issue conflicting permits, leading to a complex, prolonged approval process for community energy developers and increasing the capital costs of establishment. Rural communities face difficulties obtaining land permits and grid connection approvals due to a lack of coordination between national and regional governments. Such disconnects result in delays, inefficiencies, and conflicts in energy planning in all three countries. As an energy manager in Malawi explained, even when “*the project follows national rural electrification goals and off-grid policies*,” “*coordination with ESCOM and the DoEA can be slow when approving technical standards*” (EM8-Malawi).

In addition to the mismatch between centralised and decentralised models of grid development, implementation is hindered by limited capacity across all levels of governance. For example, in Ethiopia, enthusiasm for decentralisation is constrained by limited capacity within regional governments to ensure projects actually happen. As one interviewee remarked: “*There is a policy there, but it needs someone who can implement the policy on the ground*” (EM4-Ethiopia). There is also a time component to these challenges, and a significant gap in monitoring and evaluation to ensure compliance, leading to the perception that “*lack of monitoring leads to major damage*” (EM5-Ethiopia). In Malawi, the lack of skilled technicians, particularly when dealing with changing operational circumstances (new solar equipment, unexpected flow variations in hydropower), is a common reason for delays (as reported by both EM8-Malawi and EM9-Malawi). This challenge is exacerbated by unrealistic expectations within regulatory institutions about the complexities of the implementation processes, which appear to leave little room for context-appropriate interpretations. In Mozambique, a representative of an international organisation explained that “*The biggest challenge is capacity from both sides of government to ensure that the policy is implemented to the letter*” (EM14-Mozambique).

The community can play a central role in facilitating access to sites, particularly when negotiating land tenure challenges. Accounts from Ethiopia suggest that community members often intervene to ensure the continuity of the project when, after acceptance, the community provided suitable land: “*First, the village administration gave us land, but when we studied the site, it had too much shade. Later, one person gave us his private land, saying, ‘I don’t want to lose the project.’ The administration replaced his land so we could use this new site. Therefore, from individuals to the administration, they were very helpful in getting the suitable site*” (EM1-Ethiopia). In Malawi, community energy projects struggle to secure land rights because land tenure policies are unclear and require both local and national permits. Customary land ownership in rural areas complicates the legal framework for renewable energy development. Here is where the community can play a central role, as our case studies show that one factor facilitating success is the provision of land by the community’s chief or the village (EM8-Malawi, EM9-Malawi). In Mozambique, municipal governments often lack the authority to approve mini-grids. Land-use regulations that prioritise productive uses make it difficult for communities to secure land for renewable energy projects, even when policies support rural electrification. Lack of access to secure tenure in Mozambique directly hinders the development projects: “*We went for solar home systems because we didn’t want to have these types of conversations with the government about land*” (EM14-Mozambique).

What this suggests is that changes in the energy sector’s institutional landscape, with frameworks that support off-grid energy and, potentially, community energy, are creating new opportunities for collaboration in which implementation challenges (coordination, licensing, land access) are resolved through active communities. The challenge is the development of appropriate capacities at the regional and local level, including developing the technical expertise of IPPs, cooperatives, and communities to navigate complex regulatory requirements for energy project approvals and land access, but also the development of capacities among donors and subnational regulators to understand and support the new infrastructure models for a just energy transition.

### Making community energy happen through finance and supply chains

The database of projects shows that in Ethiopia, the CE landscape is highly homogeneous, dominated by micro-hydropower projects. In contrast, the landscape of CE in Malawi is more heterogeneous, including a broad spectrum of large hydro, solar and hybrid systems. Overall, community energy projects remain limited in their scope and coverage. Moreover, they may have a short run. In Ethiopia, 7 out of 11 projects were not running. In Malawi, 10 out of 30 were not running. Technology and capacity influence the reach of CE and the extent to which it supports local livelihoods. In general, looking at the database, hydro projects are versatile and support both domestic (lighting, phone charging) and productive uses (milling, irrigation, industry). Hybrid (Solar and Wind) systems are most often deployed for basic services, especially lighting and phone charging. Solar systems usually support water pumping, irrigation, and small-scale amenities such as radios and TVs. Since this data was compiled, we have also seen growing interest in using solar power to power hospitals and health facilities.

Table 1 provides an overview of the different financing models in each country, highlighting the dominance of national public funds in Ethiopia and ODA funding in Malawi. Off-grid energy projects in Ethiopia are traditionally government-funded and led. However, small grants are sometimes used to fund localised and diverse community energy projects, for example, in hospitals (not included in the database). The list compiled for Mozambique also shows that the government has traditionally dominated the development of off-grid projects through FUNAE’s operations. In Malawi, CE projects draw from a diverse range of finance sources, including the government, NGOs, international organisations and the private sector, which tend to fund specific projects for fixed-term periods. For example, Churches Action in Relief and Development (CARD) has financed four projects, primarily solar, but most organisations, from MERA to the civil society organisation Community Energy Malawi, fund one or two CE projects. These unique differences in funding of the development of energy infrastructure have implications for how off-grid energy systems, such as community energy initiatives, are financed. However, while the table may suggest that these financing mechanisms are independent of each other, our database shows that most projects integrate diverse sources of funding, such as grants, ODA funds, microfinance, and private-sector investment, through a blended finance framework, plus support from the community and in-kind contributions from other actors such as Universities. This approach is more financially stable than relying on a single source but requires coordination among diverse financial partners. Thus, most of the projects reviewed depend on complex partnerships involving civil society, international donors and communities. This questions the viability of do-it-yourself governance models to manage community energy^22^, in favour of complex and dynamic partnerships that adapt to the changing conditions of development.

**Table 1:** Financial models for community energy initiatives found in the case studies in Ethiopia and Malawi, and those reported by key stakeholders in Mozambique (legend √ -present, *dominant)

Financial models for the development of community energy initiatives		Case studies		
		Ethiopia	Malawi	Mozambique
Public sector and government-supported financing				
1	National rural electrification funds	√ *	√	√
2	Public-private partnership	√	√	√
Private sector and market-based financing				
3	Microfinance and pay-As-You-Go (PAYGO) models	√	√	√
4	Community cooperatives and energy associations		√	
5	Impact investing and social enterprises	√	√	
International donor and development financing				
6	Grants from development agencies and NGOs	√	√ *	√
7	Climate finance and carbon credits	√		√
Blended finance approaches				
8	Grants + microfinance + private sector investment	√	√	√

Several financing models are commonly used to support off-grid renewable energy solutions, particularly within national programmes for rural electrification (see Table 1). For example, in Ethiopia, the NEP2 and in Malawi, the Rural Electrification Fund (REF) support off-grid community energy projects. In Mozambique, FUNAE has long promoted off-grid energy to reach areas beyond the grid. New ambitious strategies for just transitions, such as the one just adopted in Mozambique, or ambitious programmes such as Mission 300 in Malawi, offer new opportunities for the concessional funds and blended finance needed to develop community energy. Thus, there is room to create a coherent financing strategy for CE. However, the focus of policy efforts so far has concentrated on leveraging investment from the energy sector with strategies to derisk investments in community energy^23^. The database of projects shows clearly that private investments in community energy are failing to materialise. There is one single project with private support, led by the Lujeri Tea Company in Malawi, but led by a social enterprise called the Mulanje Electricity Generation Agency (MEGA)^24^. Public-private partnership models and other strategies for derisking investment have not attracted the private capital needed to make CE sustainable, underscoring the importance of blended finance and concessional grants^12^.

A significant obstacle is ensuring that tariffs are affordable. Most communities have different payment systems for services. In Malawi, those with electricity meters pay a flat rate based on consumption, while those without electricity meters pay a fixed monthly flat rate. These rates are decided mainly by committee members in project Boards. In Ethiopia, most communities pay a monthly flat rate, except for one community energy initiative, which provides electricity free of charge. The interviews reveal that current tariff models do not provide sufficient funding to sustain existing projects. For example, in the Tigray project, “*the community collects and manages tariffs, but the income is insufficient [to cover running costs]*” (EM4-Ethiopia). In Malawi, a manager explains that they “*collect small tariffs, but not enough for maintenance*” (EM9-Malawi). This highlights the need for better, targeted business models to ensure adequate funding for the maintenance and operation of CE. These gaps in service provision undermine trust of communities and stakeholders that invest in these initiatives, despite evidence of their potential to support sustainable energy transitions for millions of communities across the continent. This is not due to a lack of financial innovations to make investments in small energy projects more attractive, especially with the use of mobile technologies. Microfinance and Pay-As-You-Go (PAYGO) models can support community energy systems, particularly solar home systems and mini-grids, by enabling rural households to afford them. Companies like d.light, M-KOPA, Yellow Solar, and Azuri Technologies offer PAYGO models in countries such as Ethiopia and Malawi. However positive these financial innovations are, business models are still lacking to facilitate their use to make CE financially sustainable.

Yet, the community makes a significant difference to the financing of community energy projects. Communities add labour and resources to the design, construction, maintenance and management of those projects. This may make a considerable difference in both the capital costs and the long-term sustainability of the projects. In the case study in Tigray, for example, the community “*prepared the road for free, contributed cash and in-kind, and one farmer voluntarily gave his land for the project*” (EM1-Ethiopia). In one of the projects in Malawi, the community “*helped with site preparation, fencing, and installation. They now form the management committee responsible for billing*” (EM8 Malawi). This is common across all projects: the community can cover capital costs and keep maintenance costs low over the long term. Moreover, communities may establish community cooperatives and energy associations to facilitate financing for community energy and to support the project when unexpected incidental costs arise. In the Maputo lab, for example, the Association Aerosol, formed by community members to govern the project, played a key role in keeping the project alive during the turmoil that followed the 2024 elections, including repairing equipment damaged by the riots. These governance models involve pooling resources, with communities contributing labour and, sometimes, funding to extend power lines to their homes. For instance, in Malawi, many solar mini-grids are operated under cooperative models. In Mozambique, local energy cooperatives, backed by NGOs, are on the rise. While these models promote local ownership and community involvement, many struggle to secure financing and require external technical assistance. Impact investing and social enterprises involve private investors funding socially beneficial energy projects for modest financial returns. In Malawi, impact investors back social solar energy enterprises, while in Ethiopia, off-grid solar businesses receive support from social impact investors. This model offers long-term investment for community projects, but it is not yet well-established in Africa.

The participation of social impact investors depends on the recognition of the social benefits that community energy provides. Social impact justifies the support of concessional grants at the national level. This is also a strong motivation for ODA programmes, which have already played a strong role in supporting CE. For instance, in Ethiopia, organisations such as the World Bank, the African Development Bank, GIZ, and UNDP fund energy access initiatives. In Malawi, projects for rural electrification are supported by the Global Environment Facility, UNDP, and USAID. In Mozambique, off-grid solar projects are funded by the World Bank and UNDP. The multilateral system, however, struggles to support CE. Some projects have explored accessing climate finance, but the small size of CE projects makes such access difficult. In this context, a re-evaluation of the social impact of CE projects may help identify alternative ways to value and finance them.

In addition to funding, regional and local governmental institutions may also play an enabling role in project delivery, and sometimes their intervention is sufficient to derisk investments. Moreover, the intervention of public institutions inserts CE in wider programmes for local and rural development. For example, the Sidama project in Hawassa involves GIZ and the local Sidama Development Association, but “*the district energy office provides monitoring and technical support*” (EM6-Ethiopia). In Chipopoma, Malawi, “*alongside Mzuzu University, the Energy Regulatory Authority provides technical support and policy guidance*” (EM9-Malawi). In Maputo, the community laboratory in the neighbourhood of Aeroporto B was made possible by the municipality’s punctual intervention, which provided reassurance and coordinated partners in collaboration with Eduardo Mondlane University of Maputo. The role of public institutions in facilitating continuity is vital, and they can also impede the projects. In Tigray, for example, “*coordination was difficult because regional authorities were unsure of our capability to develop and implement the project*” (EM2-Ethiopia). In Malawi and Mozambique, where the sector is strongly dependent on ODA, the lack of coordination between donor-funded programming and the development of energy regulations is seen as a significant obstacle to finance (EM10-Mozambique). In summary, Table 1 presents the variety of financial models reported during the visits.

In terms of supply chains, Africa has abundant reserves of crucial minerals, such as lithium, graphite, cobalt, and copper, which are essential for clean energy technologies. These minerals are key parts of products like lithium-ion batteries, which are necessary for energy storage solutions. Africa currently generates over USD 20 billion annually from copper and significant production of battery metals^25^.

Despite this wealth, the continent’s role in the manufacturing and assembling of renewable energy technologies remains limited, making it wholly dependent on imports^26,27^. Lack of access to advanced technology and technical knowledge hinders the continent’s progress in transitioning from raw material extraction to manufacturing. Empirical data show that technologies used in the development of community energy systems are almost always imported, affecting the time scale of project implementation and project costs. For example, community energy projects in Malawi often import generators and turbines, and projects that have tried to develop local skills to build turbines have eventually replaced them in the long run (as happened in the case of Chipopoma).

Inadequate infrastructure, along with unclear policies and regulatory environments, is further discouraging investment and stalling the growth of local industries. Most investment in renewable energy focuses on importing technology rather than building local manufacturing capacity, leaving supply chains for off-grid systems vulnerable to disruptions^28,29^ Such challenges result in higher project costs and delays in implementation. Relying on imported technology also makes it difficult to procure spare parts, resulting in more extended downtime during system servicing. For example, solar technology for a project in the Tigray region of Ethiopia faced delays of over six months at customs due to security clearance for radio wave meters. In Ethiopia, installing solar systems at power clinics took around eight months due to various challenges, including obtaining import licenses, securing bank approvals for foreign currency, and arranging sea and land transportation. In Mozambique, the construction of a community energy lab faced significant challenges when importing technology, conditioning the available technologies and the project timeline, and forcing the team to build amid uncertainty amid the contested October 2024 elections.

### Contributions of community energy for just transitions

Community energy’s outcomes often target specifically marginalised or vulnerable groups, such as those suffering discrimination due to gender, place of origin, race, level of ability, or sexual orientation, which would not be reached otherwise^11^. For example, community energy projects have greater potential to deliver gender equality outcomes than larger electrification projects because beneficiaries have more opportunities to influence decision-making and shape the project’s benefits. They also provide opportunities for women to develop engineering skills in practice, helping bridge the gender gap in technical skills in the energy transition. Some community energy projects foster reciprocity and solidarity, thereby strengthening social capital and building the community’s resilience.

Like mini-grids, community energy initiatives create opportunities for new businesses like milling, barbershops, bars, and restaurants, as well as access to updated information through TV and radio. They also enable children to study at night. A similar study conducted in Kenya showed that community energy solutions lead to enhanced productivity and improved delivery of both social and business services^30^. The advantage of CE projects, however, is that they enable close tailoring to the needs of communities, and community commitment sustains these projects through complex challenges. Moreover, community energy projects specifically build the community’s human, economic, social, and physical capital, plus well-being, enabling it to cope with any unforeseen disruptions with minimum external support^31^. Table 2 summarises community energy benefits documented in the literature.

**Table 2:** Community energy benefits^6,44^

Community energy benefits	
Economic	Energy Access/last-mile access to electricity Financial benefit for the community Services for marginalised areas or communities Higher employment Social inclusion Support for other community activities & services, such as maize mills, oil extractors, etc. Savings on expenditure on fossil fuel, such as by using electric water pumps for irrigation
Education and acceptance	Knowledge about energy-saving Understanding how to run community projects Examples that can inspire other communities Improving trust and acceptance towards renewable energy
Participation	Higher level of political participation Collective financial management Self-organisation
Climate protection and sustainability	Awareness and lifestyle changes Provision of a low-carbon supply of energy Reduced deforestation
Community building and self-realisation	Community upgrading Social cohesion and more robust governance Pride, joy, and other emotions related to collective material action
RE generation targets	Increase the share of renewables in the energy supply Level the playing field for market entrants Building supply chains to facilitate RE adoption
Technical Skills and Innovation	Engineering systems design skills Power systems operation, monitoring, and troubleshooting skills Endogenous innovation Generation of new societal norms
Infrastructure	Power generation and distribution infrastructure fitting the conditions of the community Communication with the rest of the world through television and mobile telecom channels Tailored or portable healthcare facilities through refrigeration of vaccines and life-saving drugs

In Malawi and Ethiopia, beneficiaries may not necessarily identify the wide range of benefits provided by CE. Household surveys may be inappropriate tools for exploring the intangible benefits that make CE an ideal contributor to the democratisation of local governance and community well-being. Even so, local households have a positive outlook towards community energy, driven by specific material benefits (Table 3). The table, however, shows that priorities may vary widely across contexts. Community energy beneficiaries in Malawi identified three benefits on average, while beneficiaries in Ethiopia identified 4 or 5 on average. Taking that into consideration, benefits such as lighting and security, education, and providing public services are priorities for more than half of the households in both countries. However, other benefits, such as information/media and health, are more commonly mentioned in households in Ethiopia, while business/income is a more substantial concern in Malawi. The surveys show that CE play different roles in different locations. Still, in each case, access to energy becomes essential for people because of the material services it provides, while intangible benefits are more difficult to identify. When explicitly asked, over 50% of beneficiaries in the projects in Ethiopia reported that the projects supported disadvantaged groups, with about 30% mentioning support for women and 11% noting support for people with disabilities or the elderly. In Malawi, however, 68% of beneficiaries did not specify whether disadvantaged groups benefited from CE, although 50% agreed that CE addressed vulnerabilities. As in the Ethiopian dataset, about 12% of interviewees mentioned benefits for people with disabilities, and 34% mentioned benefits for women.

**Table 3:** Keywords mentioned by interviewees in household interviews

Keywords mentioned	Mentions (Malawi)	%	Mentions (Ethiopia)	%
business/income	219	62	83	55
lighting/security	207	59	119	79
education/study	188	54	94	62
community/public services	168	48	122	81
information/media	88	25	95	63
time savings/convenience	81	23	58	38
savings/cost reduction	66	19	34	23
health	30	9	94	62
comfort/quality of life	27	8	3	2
TOTAL INTERVIEWEES	**351**		**151**	

Limited capacity, frequent power outages (for example, during dry seasons due to low water levels in hydro-based energy systems), and inadequate maintenance hinder the potential of community energy to deliver these benefits. Despite these challenges, the household survey suggests that households perceive that these energy projects have significantly improved their quality of life.

## Discussion

There is a need for a conversation about the kind of governmental support that can make a difference in the development of renewables in each country or location^17^. Historical and quantitative assessment of the governance of energy access demonstrates that an appropriate regulatory framework is necessary but not sufficient to develop community energy at scale. CE depends on establishing dynamic, flexible partnerships that respond nimbly to a changing energy development context. The experiences of managing community energy suggest that decentralisation facilitates community energy development. For example, Community Energy Malawi (CEM) piloted a District Energy Officers programme that built capacity for energy planning and has facilitated the rapid growth of microgrids. In addition, various other social actors foster community energy and could benefit from additional support. In Ethiopia, for example, religious institutions may act as community-dynamising institutions, facilitating the inception of community energy projects.

Governments can also implement measures to maintain supply chains and promote community energy development, given the reliance on imported technology and resources, as we found in all case studies. Tax exemptions encourage the development of off-grid renewables. They have been widely applied in Malawi and, to a lesser extent, in Ethiopia and Mozambique. However, an energy transition will require developing endogenous technological resources and fostering industrial capacity. In addition, circular economy policies to facilitate the reuse and recycling of materials and technologies (such as initiatives to recycle batteries in Mozambique) can help address supply chain challenges. Investing in manufacturing capabilities for renewable energy technologies can create jobs, reduce costs, and build resilience against global supply chain disruptions. The South African Renewable Energy Masterplan emphasises the importance of evaluating local manufacturing capabilities through a value-chain approach, a crucial first step that other African countries should follow to develop and strengthen domestic industrial capacity^32^. Collaboration among African nations can create regional value chains, maximising resource use and market reach.

The discussion invariably returns to financing and how to make the social benefits of CE investable. Capital investment has always been central to the development of community-led renewables, irrespective of their geography^9^. Current levels of investment are not appropriate to sustain a sufficiently large network of community energy projects to facilitate an energy transition. Most funding comes in the form of limited-time grants for individual projects. There is a need to finance relatively small projects, emphasising stability, for example, by providing contingency funds for shocks and service disruptions.

Donors could help develop a dynamic financial landscape to integrate community energy projects into the energy transition. The most effective forms of financing rely upon long-term partnerships between donors and service providers, enabling them to share financial costs and burdens over time. Donors could emphasise, for example, the social return on investment, a method to account for a wider range of values than those considered in traditional financial statements, including social, economic and environmental benefits such as those provided by community energy. Additionally, implementing favourable policies and incentives can attract both domestic and foreign investment into the energy sector.

As renewable projects have grown, there has been a shift towards greater private-sector participation and non-concessional funding^33^. However, this shift is detrimental to the development of community energy because, despite generating clear material social benefits, these projects are not always attractive to private investors. Dedicated funding programs that emphasise speed of response rather than large amounts of capital could make a difference for communities eager to deploy their abilities in the energy transition. At the core of CE, communities provide resources and labour, reduce costs, and integrate new technologies into their lives. Their role in facilitating a just transition to clean energy is invaluable.

The benefits of CE cannot be understated. Descriptive evidence from surveys demonstrates that community energy directly addresses the needs of marginalised or vulnerable groups that would not be reached otherwise, even when suffering discrimination due to gender, origin, race, level of ability, or sexual orientation. By targeting these groups, community energy can enhance energy access and support a just transition to cleaner energy sources that reach those most disadvantaged. The most important finding is that communities value the material benefits provided by community energy, even when the projects serving them faced practical challenges. These material benefits should be central to recognising the social value of CE, thereby facilitating the development of innovative financial models to support small, place-based energy projects.

From the perspective of a just energy transition, CE can make a significant difference to the adoption of renewables across society, catalysing institutional change and creating new ways of thinking about the energy transition^34^. The development of community energy projects at scale requires the support of a wide range of regional and local institutions that can mediate the gap between global opportunities and local implementation. Still, capacity and skills need to be improved at all levels. This argument can also be reversed. CE enables technicians, policymakers, and users to develop the skills needed to accelerate the transition to sustainable energy. The incorporation of communities with their specific local experiences, capacities, and abilities can accelerate this process and help fill the labour gap. Above all, however, community energy gives a voice to those whose views and experiences are overlooked, and this is one of the surest ways to advance a just transition. As we finish writing this paper, UNEP has published a new Global Environmental Outlook (GEO-7), arguing in no uncertain terms that^35^:

“*Equitable benefit-sharing together with participatory and inclusive decision-making for disadvantaged and marginalized groups, including Indigenous Peoples, can help prevent new energy developments from perpetuating harms and injustices*”(p. 810).

## Methods

This paper is the result of a collaborative multi-institutional research partnership funded by the UK Global Challenges Research Fund to explore the potential of community energy systems to accelerate inclusive, just, and clean energy transitions in Ethiopia, Malawi, and Mozambique. Learning from previous work on community energy by the International Renewable Energy Agency (IRENA)^36^, we defined community energy as heterogeneous systems involving technological solutions ranging from mini-grids to localised solar for community infrastructure.

The working hypothesis is that benefits ensue when communities play a key role in making community energy:

- In decision-making: communities must direct decisions about the development of energy systems. They often constitute decision-making bodies, such as Boards, to facilitate their management.
- In maintenance and sustainability: communities perform work to maintain and repair infrastructure over time and anticipate the project’s threats, such as flooding or lightning.
- In needs and inclusion: communities build the knowledge base that informs the operation of community energy systems, assessing the needs of people in the community and identifying vulnerable groups or individuals whose needs may require focused attention.

The design of the methodology departed from the idea that communities are not homogeneous: diverse perspectives, sometimes conflicting, and power relations hold them together. Moreover, communities are not isolated. Community energy projects depend on the broader networks that influence communities, especially the political and economic institutions that shape their livelihoods and the opportunities they can access. Looking at the relationship between community energy and the possibilities for a just energy transition, we formulated operational research questions as outlined in the introduction.

### Research design

Existing research on community energy tends to compile data on operations, such as energy output, participant numbers and financial performance, but this does not facilitate the study of the fit between the project and its context, community dynamics, and the multi-level impact of political economy factors. A comprehensive literature review highlights that while many evaluations report operational metrics, they typically fail to address the interplay between community energy projects and their broader socio-political and economic environments^37^. Similarly, a recent multi-level policy analysis showed that misaligned governance structures across local, regional, and national scales can substantially hinder community energy initiatives, a factor rarely captured when studies concentrate solely on operational outcomes^38^.

Alternatively, other research focuses on concrete case studies, such as those from Europe and North America, which provide rich qualitative insights into specific community initiatives, but the lessons remain anecdotal and have so far not transformed thinking on community energy and its role in transitions^39,40^.

In this project, we adopted a threefold, multi-method strategy to examine community energy at the national level. First, we selected three cases of study that provided a unified problematic (facilitating energy access in a context of significant gaps) but within different conditions:

- The energy sector in Ethiopia is driven by state policies and interventions, and with a strong infrastructure-delivery drive. The last 20 years have seen rapid progress in electricity access rates, particularly in urban areas (which account for over half of the population).
- The energy sector in Mozambique has undergone a rapid transition from state-led delivery to the greater intervention of the private sector and liberalisation of energy services. After stagnation, the last five years have seen optimistic advances towards SDG7 (over a quarter of the population now has access to electricity).
- The energy sector in Malawi is heavily dependent on international aid, and there is a strong presence of NGOs and community-based organisations in energy delivery. The country presents one of the slowest rates of progress towards achieving SDG7 in the world.

Regardless of these different situations, the three countries face the same problems in advancing the deployment of renewables and clean fuels and reaching remote communities, as well as communities left behind, even though they may remain in proximity to electricity networks, such as populations living in informal settlements.

In each country, we aimed to study three aspects of the community energy landscape: context, project, and community. The approach was comparative and used multiple methods:

- Understanding the political economy context for the energy transition in each country: in each country, we proceeded through a thorough review of the evolution of energy policies over sixty years (1956–2022) and how community energy has been included and with what impact.
- Understanding the landscape of current community energy in each country, which included a compilation of all existing and planned projects in each country in country databases of community energy projects, and the selection of several case study projects. We established partnerships that enabled a deep understanding of the material dynamics of each project, and, where appropriate, we conducted in-depth interviews with project managers and other stakeholders.
- Understanding the communities that benefit and govern community energy projects through qualitative research with community energy officials, a survey of community energy users, and action research with ongoing projects.

### Evidence-base and data collection

As explained above, the data collection strategy followed three areas of work: the political economy of energy transition, the landscape of community energy projects and understanding the community.

To study the political economy of energy transition, the team began by reviewing published and grey literature on energy transition in each country, including international reports and electrification assessments (from the UN SDG reports to national and regional assessments). The review included secondary sources such as academic papers, policy publications, and laws and regulations from national and international authorities. As part of our systematic review of secondary data, we examined 23 official documents from Ethiopia, 17 from Malawi, and 32 from Mozambique, encompassing regulations, proclamations, resolutions, and decrees. This constituted the starting point for developing a database of existing policies and plans for each country, as well as stakeholder mapping for each country.

**Fig. 2:**
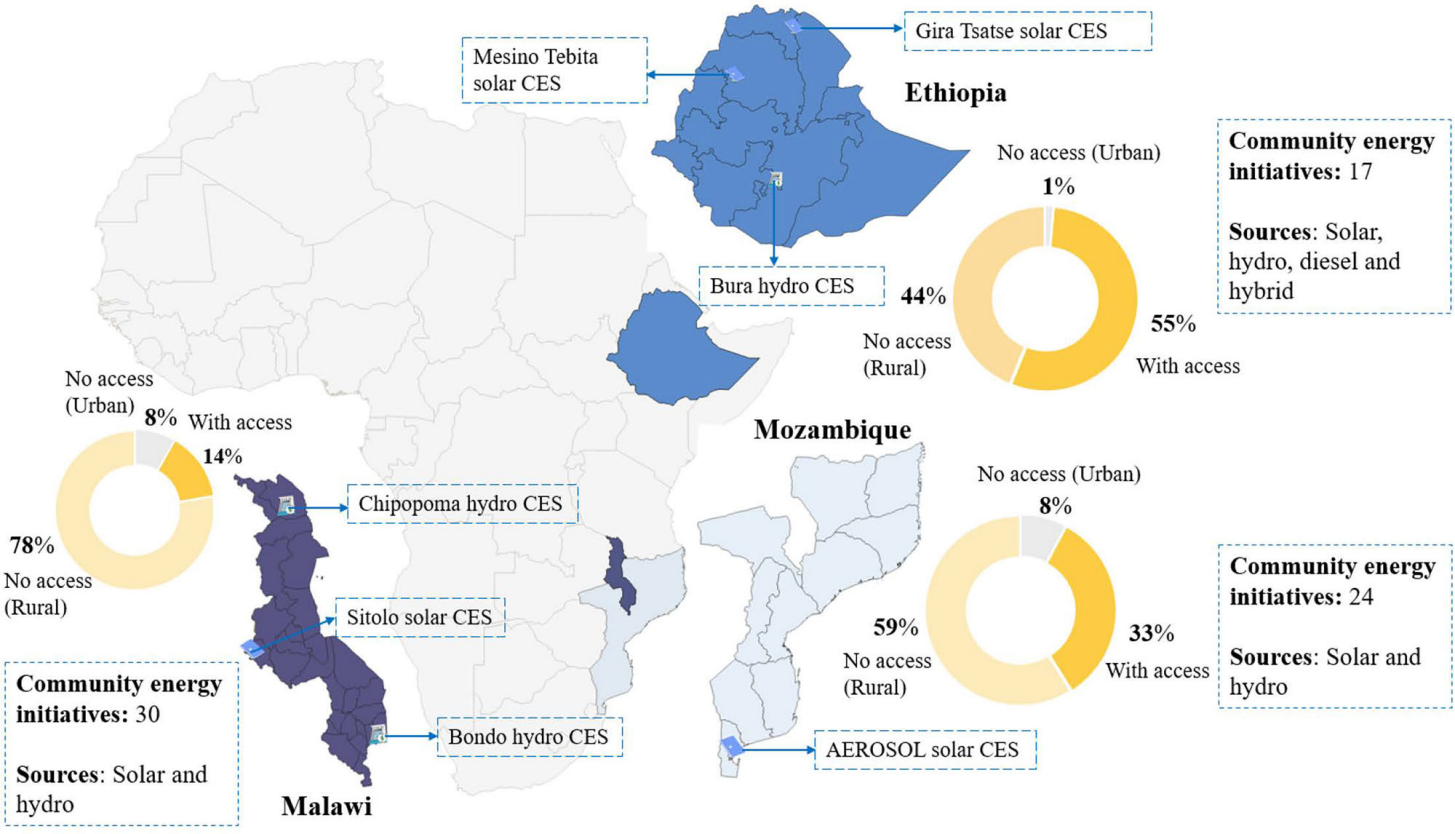
Geographical distribution of empirical material and electrification context. The figure presents the relative geographical locations of Ethiopia, Malawi, and Mozambique in Eastern and Southeastern Africa, based on data from the World Bank Development Indicators Database (own elaboration). Pie charts illustrate national electrification rates, distinguishing between the share of the population with and without access to electricity in rural and urban areas. The figure also summarises key information from the census of community energy projects identified in each country.

To study the current landscape of community energy, we compiled data on all known community energy projects in each country and completed the databases in 2021. For their inclusion in the list, the projects must meet three characteristics: (1) the project provides electricity off-grid; (2) the project has been implemented and has an identifiable set of beneficiaries; (3) it provides a collective, rather than a single family (e.g., bigger size than solar home systems). In total, we compiled 11 projects in Ethiopia, 30 in Malawi, and 65 planned in Mozambique, of which only 24 seemed to be functional (Table 4 and Fig. 2). The list was compiled through consultation with energy providers and relevant energy organisations in each country. Verification was achieved by consulting selected projects within the list and through project visits.

**Table 4:** Summary of the database of CE projects (Data collected from 2021-2023)

	Number of projects	CES size/Capacity (kW)	Average CES size/Capacity (kW)	Direct beneficiaries (number of households)
Ethiopia	17	233	21.2	5092
Micro-Hydropower	10	212.5	21.2	4892
Solar	1	20.5	20.5	200
Non-functional	6	0	0	0
Malawi	30	9859.6	616.2	1950
Hybrid (Solar and Wind)	6	80.0	20	600
Hydro	9	8638.0	1727.6	950
Solar	13	1141.6	163.1	400
Mozambique	65 (only 24 active)	2009.0	30.9	4368
**Grand Total**	**106**	**12101.6**	**131.5**	**11410**

One challenge was to assess the representativeness of the list compiled for Mozambique, which was collated in collaboration with the National Energy Fund (FUNAE). During verification, we realised that many projects had never been implemented. From these lists, six case studies were selected for further investigation. Field visits, informal interviews with project managers, and transect walks were used to construct the natural histories of projects as they unfolded: two in Ethiopia and two in Malawi. In Mozambique, however, field visits suggested that community energy was not a reality. For example, during a visit to the photovoltaic project in Calanga, led by FUNAE, we verified the installation but could not identify a community of beneficiaries.

Working closely with the managers and staff of the selected projects for case studies, the team developed an in-depth understanding of each project’s dynamics. Qualitative interviews with project managers and stakeholders were conducted to capture experiences shared across many meetings and bring them together into the stories told about each project. Local teams adapted the interview guide and translated it into the relevant languages. Some of these stories were brought to life by the project managers themselves, who wrote a chapter for the team’s book^24^. In Malawi, the team conducted informal interviews, as its members are deeply involved in energy-sector policymaking. In Mozambique, where the team struggled to find projects already running, the team conducted interviews with key actors experimenting with and building partnerships for off-grid development (Table 5). Only one of 17 interviews was with a woman. This reflects the situation in the renewable energy sector, where there is an enormous gender gap. Currently, our team has turned to that challenge as the next frontier of research in the energy transition.

**Table 5:** List of interviewees participating in qualitative interviews (anonymised)

Name	Position/Role	Affiliation/Institution	Location/Region	Gender	Project/Site
Ethiopia					
EM1-Ethiopia	Director	Tigray Regional Energy Agency	Enderta Woreda, Girar Tsetser village, Tigray	Male	*Girar Tsetser Solar Mini–Off Grid Project*
EM2-Ethiopia	Senior Energy Manager	Tigray Regional Energy Agency	Enderta Woreda, Girar Tsetser village, Tigray	Male	*Girar Tsetser Solar Mini–Off Grid Project*
EM3-Ethiopia	Energy Officer	Tigray Regional Energy Agency	Enderta Woreda, Girar Tsetser village, Tigray	Male	*Girar Tsetser Solar Mini–Off Grid Project*
EM4-Ethiopia	Senior Energy Officer	Regional Government Office	Enderta Woreda, Girar Tsetser village, Tigray	Male	*Girar Tsetser Solar Mini–Off Grid Project*
EM5-Ethiopia	Energy Manager	Sidama Regional Energy Office	Hawassa, Sidama Region	Male	*Micro-hydro Off-grid Energy Project*
EM6-Ethiopia	Energy Manager	Sidama Regional Energy Office	Hawassa, Sidama Region	Male	*Micro-hydro Off-grid Energy Project*
EM7-Ethiopia	Local Government Representative	District Job Creation Office	Hawassa, Sidama Region	Not stated	*Micro-hydro Off-grid Energy Project*
*Malawi*					
EM8-Malawi	Energy Engineer	ESCOM/Practical Action (lead partner)	Nsanje District (Nyamvuwu & Chimombo Villages)	Male	*EU-funded Community Energy Service (CES) Project – four minigrids (Chimombo, Nyamvuwu, Oleole, Mwalija)*
EM9-Malawi	Director	Chipopoma Hydro Project/Community Initiative supported by Mzuzu University & Malawi Energy Regulatory Authority	Rumphi District (Mkandapasi Village)	Male	*Chipopoma Micro-Hydro Project*
Mozambique					
EM10-Mozambique	Policy Advisor	SNV Mozambique	Maputo	Female	BRILHO
EM11-Mozambique	Policy Advisor	British High Commission, Maputo	Maputo	Male	BRILHO
EM12-Mozambique	Energy Manager	KfW/GIZ (German Development Cooperation)	Maputo (Online)	Male	N/A
EM13-Mozambique	Director	Source Capital/Source Energia	Maputo	Male	N/A
EM14-Mozambique	Energy Adviser	International Organization for Migration (IOM) Mozambique	Maputo (Online)	Male	N/A
EM15-Mozambique	Country Director	SolarWorks! Mozambique	Maputo	Male	N/A
EM16-Mozambique	Country Coordinator	GET.invest Mozambique (GIZ/EU-funded Programme)	Maputo (Online)	Male	N/A
EM17-Mozambique	Head of policy	African Development Bank (AfDB)	Maputo (Online)	Male	N/A

In addition to the qualitative interviews with a wide range of community energy experts, we also aimed to capture local perspectives from beneficiaries. Again, focusing on the four case studies in Ethiopia and Malawi, the team administered structured questionnaires to 502 beneficiary households (Table 6). The questionnaires were brief and explored the benefits of the project to people’s lives, the main perceived obstacles, the current governance arrangements, the perceptions of exclusion, and the perceptions of systems resilience (see Supplementary Information). Local teams adapted the questionnaires into the regional contexts and translated them as appropriate.

**Table 6:** Household surveys in Ethiopia and Malawi

CES Site	Male	Female	Not specified	TOTAL	% with electricity	Average Age (years)	Average Household Members (n)
MALAWI							
Bondo	70	86	0	156	97	41.3	4.6
Chimombo	14	2	0	16	93	38.1	5.2
Mantchewe	13	20	0	33	100	37.3	5.1
Mnthembanji	15	18	0	33	100	36.9	5.8
Nyamvuwu	13	1	0	14	85	42.8	5.3
Sitolo	46	53	0	99	95	41.3	4.7
TOTAL MALAWI	**171**	**180**		**351**			
Ethiopia							
Gira-Tsatse	27	72	1	100	100	48	6.09
Mesino tebita	5	20		25	100	n/a	n/a
SIDAMA		1	25	26	100	n/a	n/a
TOTAL ETHIOPIA	**32**	**88**		**151**			

Finally, an essential source of data was our engagement in action research projects in each country. In Ethiopia, the team works directly with a team delivering community energy in hospitals in Tigray, supporting the installation of PV with resources and technical expertise. In Malawi, we work with two relatively consolidated projects (the Chipopoma Project in Livingstonia and the Mulanje Energy Project), supporting them in developing a more effective tariff system and providing support during shocks (such as lightning) (one of them also included as a case study). In Mozambique, we have constructed an ‘energy laboratory’ from scratch to palliate the difficulties of studying community energy in that context. The community engagement process lasted 18 months and was mediated by the municipality, with weekly meetings and an open tendering process to deliver the project. The local community has formed an association that now owns the project, known locally as Aerosol. Despite local enthusiasm, the project was delayed and almost collapsed after the violence following the October 2024 elections, but it was finally completed in November 2025.

During our research activities, we adhered to the ethical principles of the participating organisations, including voluntary participation and informed consent. We received participants’ consent to use this data, subject to anonymisation practices for personal data.

### Data analysis

To understand the political economy of energy, background data on the context were compiled through historiographies of energy policy in each country, which served as a point of reference to explain the context. These historizations were presented to colleagues active in the energy sectors of each country, with emphasis on their implications for transitions and on the sparse information relevant to the community energy directly. Specific analysis of community energy definitions in existing policies and identifiable barriers was conducted. This analysis was complemented by a qualitative thematic assessment of expert opinions compiled through qualitative interviews, which helped systematise these barriers. Themes explored were policy coherence, implementation challenges, licensing, land access, partnerships, financing, supply chains, community involvement and governance. In addition, the analysis explored the relationship between the development of policy and its outcomes, with the statistical evaluation of hypotheses about the influence of different coded characteristics of energy policy (pioneering character, density of regulations, mentions of community energy) on measurable outcomes (rates of electricity access, number of community energy projects).

To understand the landscape of community energy, the data on projects was analysed comparatively, seeking to establish patterns of operation and verify how the different barriers applied in each case. An important strategy was the systematic analysis of failure in community energy projects, examining individual project histories through process tracing to verify key barriers and systematise their impact. Within projects, we combined field visits and action research data with a systematic thematic analysis of managers’ perspectives. This was consolidated into a perspective from the ground, which could be compared with the study of the implementation context.

Secondary data on the evolution of policies across the three countries were analysed to identify their benefits for community energy projects and map them across the different contexts. The team used a method called Most Similar System Design (MSSD) to compare countries that share standard features, to identify key differences influencing outcomes^41^. This technique, as described by Przerworski and Teune^42^, is ideal for area studies of Eastern Africa, which encompasses countries with diverse histories, languages, religions, politics, and cultures. By focusing on similarities across geographical regions, MSSD helps uncover significant variations within similar contexts, aiding understanding of outcomes in Eastern African countries. This led to the development of a comprehensive list of barriers and implications on community energy developments as described in the three results sections above.

An additional layer of information was provided by the action research projects on community energy, which offer insights into the actual practice of community energy. This kind of knowledge embedded in practice is sometimes referred to as ‘phronesis,’ that is, the kind of wisdom that develops from practising expertise^43^. These experiences enabled realistic readings of systematic knowledge in relation to project delivery, as well as an understanding of the dynamics of community engagement and its pitfalls.

The study of community energy is fraught with difficulties because there is no systematic data that engages with its multi-layered dimensions. As the account above demonstrates, our project encountered multiple challenges in engaging with the different dimensions of community energy due to a lack of data or data that was simply inaccurate (as in the case of Mozambique). The challenge of the energy transition, however, is to move from studying what we know to open avenues to understand how future pathways can be facilitated. It is with this aim that we offer a methodology fully engaged in the context study, albeit limited, to prove beyond doubt the suitability of community energy. We expect that this study will help generate specific hypotheses to be tested in more confined settings in future research.

## Supplementary information

Supplementary Information

## Data Availability

The data is available on request and will be deposited in the Datareshare repository in 2026 (giving a reasonable time after the completion of the project to publish its results).
