# Supplementary material for: The role of community energy in mediating sustainable energy transitions in East and Southern Eastern Africa
Source: Npj Clean Energy. 2026 May 20;2(1):10. doi: 10.1038/s44406-026-00024-w (PMC13218297; doi:10.1038/s44406-026-00024-w)
Supplement: Supplementary file 1 — Supplementary Information [file 44406_2026_24_MOESM1_ESM.docx]

**INTERVIEW GUIDE 1: CESET COMMUNITY ENERGY HOUSEHOLD QUESTIONNAIRE**

**To be adapted to the specific context in communities in Ethiopia, Malawi and Mozambique, which have access to community energy projects**

This research Questionnaire is prepared to collect data to understand the energy landscape in Ethiopia, Malawi and Mozambique. The data will be used for academic purposes as well as by local, national, and international stakeholders interested in the development of community energy. Thus, we kindly request your support in completing the questionnaire and being part of the solution to address the various issues surrounding community energy projects.

**Note:** Please follow the informed consent procedure and explain CESET policies for anonymisation, confidentiality and data protection.

**Demographic data**

| **Region** |  | **District** |  |
| --- | --- | --- | --- |
| **Interview ID** |  | **Gender** |  |
| **Age group (under 18, 18-34,35-64, 64 and over)** |  | **Name of the project** |  |
| **Number of members of the household** |  | **Additional information** |  |
| **HH Connection (Y/N)** |  | **Connection Duration** |  |

**QUESTIONS ABOUT THE HOUSEHOLD**

1. What are your energy needs, and how are they met?

Prompts:

- 1. What fuels do you use and for what uses (*e.g. fuels: electricity, charcoal, kerosene, LPG, CNG, oil, firewood, animal dung; e.g. uses: cooking, transport, productive, other*)? Where do you get them from? (*e.g., collection, purchase from wholesalers or retailers, donors*)
  2. What do you use electricity for? Where do you get electricity from? (e.g. )

1. Can you estimate what percentage of household income is used for fuels and for electricity?
   1. What is the source of income? (*e.g., Casual Labour/Salaried Employment/Self-employed business/ commercial farming/ other*)

**BENEFITS FROM THE COMMUNITY ENERGY PROJECT**

1. *Can you explain how the community energy project benefits you?*
   1. *Personal benefits*
   2. *Benefits to the household*
   3. *Public uses (e.g., schools, public facilities)*
   4. Productive uses/uses to make a living (*e.g., industrial uses, commercial uses*)
2. Are there any challenges in accessing and benefiting from the community energy project?

**HISTORY OF THE COMMUNITY ENERGY PROJECT**

1. Can you describe how you joined the community energy project? Do you know who started it?
   1. What kind of key events have shaped its development? For example, has the project been affected by natural disasters? By political events?
2. What do you think made it possible? (*e.g., donation, institutional leadership*)
   1. What have been the main difficulties faced by the project?

**GOVERNANCE AND MANAGEMENT**

1. How is the system managed and maintained?
   1. Who is responsible for day-to-day management?
   2. Who is responsible for repair and maintenance and how does it take place?
2. Are you involved in any decisions about the community energy project? Can you explain what forms of engagement you have and how it happens?
   1. What actions could help you to be more involved?
3. How are tariffs established and how do they work for users?

**DIVERSITY**

1. To what extent does the community energy project address the needs of vulnerable groups (*e.g. women, children, elderly, disabled people, people from minority ethnic groups?*)
   1. Do members of vulnerable groups participate in taking decisions about the project? How?

**CONCLUSION**

Thank you so much for your valuable contribution to our research

Do you have any comments or questions?

**Thank you**

**INTERVIEW GUIDE 2: INTERVIEW GUIDE FOR MANAGERS/PROJECT OFFICERS**

**To be responded by project managers/project officers and other stakeholders implementing community energy projects in Ethiopia**

This interview guide focuses on the energy landscape in Ethiopia: what facilitates or prevents community energy projects. The data will be used for academics as well as to **local, national, and international stakeholders** who are interested in the development of community energy. We kindly request your support to participate in this interview and be part of the solution to address the different issues around community energy projects.

**Note:** Any information provided by respondents will be kept confidential.

| **Region** |  | **District** |  |
| --- | --- | --- | --- |
| **Village** |  | **Gender** |  |
| **Name of Respondent** |  | **Role or Position** |  |
| **Name of Project** |  | **Project Duration** |  |

**Energy Governance**

1. To what extent does existing energy policy support community energy projects, in your opinion?

- *What policy aspects support off-grid development?*
- *What aspects of energy policy hinder off-grid development?*
- *Are there policy gaps to support off-grid development?*

1. Can you give an account of how this community energy project came about?

- *What are the key characteristics of the community energy project? What services does it provides? To how many households, businesses, others?*

1. What institutions oversee community energy delivery and what are their roles?

- *How was the project developed? Who are its partners? What brought them together?*
- *Was the local community consulted prior to approval of the off-grid energy system development? How?*

1. How are the communities/end users involved in the energy project?

- *In what ways are they involved? At what stage? (For example, communities can be involved in the design/layout, in planning, and in the governance of the community energy project)*
- *To what extent do communities influence the community energy project?*
- *Are women involved in the community energy project? How do you involve them?*
- *Are there other disadvantaged groups involved in the community energy project? How?*
- *Is there anything that would prevent people from participating in the community energy project? (e.g. age, ethnic group, gender, having a disability)*

1. Are there local community-based social structures that influence/support the development of off-grid projects in your community?
2. How did you assess the energy needs of the community?
3. What kind of business model does this project use?

- *How did you come up with this business model and does it fit the current situation?*
- *Is it effective? Are there any challenges?*

1. How was land secured for the community energy project?

- *How was the land acquired?*
- *Were there land-related challenges?*

1. What challenges did you face during setup and installation of the community energy project?

- *Possible challenges include challenges in the conception of the project, access to technologies, lack of institutional support, local disputes, lack of capacities, maintenance issues, etc.*
- *How did you manage these challenges?*

**Energy Flows**

1. What are the kind of energy resources used by households not connected to the community energy system?
2. What are the positive impacts that this energy project has brought to the community?

- *To the households connected to the energy system*
- *Households not connected?*

1. Do you feel that the development of the community energy system has helped to change the energy patterns of this community?

**Energy Choreographies**

1. Is the off-grid system properly integrated into the current infrastructure and housing structures?

- *How was it integrated?*
- *Any difficulties?*

1. Are there any challenges for new entrants of community energy technologies?

- *How are they managed?*

1. Have there been any events that caused disruption to the community energy system?

- *What happened and when?*
- *What helped to manage this disruption? How did the community help?*

1. How does the community energy project address different needs from the population, in particular, the needs of those who may be more vulnerable?

- *What aspects of people’s vulnerability are considered?*
- *For example, to what extent the project considers the needs of:*
  1. *Women*
  2. *Young people and the elderly*
  3. *People with disabilities*
  4. *People with the lowest incomes*
  5. *People from different ethnic groups or different origin than the majority of the community*

1. What are the future plans for the community energy project?
